# Lymphedema and employability – Review and results of a survey of Austrian experts

**DOI:** 10.1007/s00508-017-1167-1

**Published:** 2017-01-26

**Authors:** Markus Neubauer, Dieter Schoberwalter, Fadime Cenik, Mohammad Keilani, Richard Crevenna

**Affiliations:** 1 Department of Physical Medicine and Rehabilitation, Medical University of Vienna, Waehringer Guertel 18–20, 1090 Vienna, Austria; 2 Department of Cardiology, Hanusch-Krankenhaus, Vienna, Austria

**Keywords:** Lymphedema, Employability, Return to work, Work demand

## Abstract

**Background:**

Literature about lymphedema and its influence on the ability to work and employability is limited. The aim of the present study was to investigate the opinion of Austrian experts on factors influencing the ability to work and employability in patients suffering from lymphedema.

**Methods:**

A self-administered questionnaire consisting of 6 questions was sent to 12 Austrian lymphedema experts with 6 different specializations from May to August 2016. These experts were asked about suitable and unsuitable professions, the possible influence of lymphedema on the ability to work and employability as well as about existing and additional measures to improve the return to work.

**Results:**

The reply rate was 100% (12 out of 12). All experts agreed that lymphedema can restrict the ability to work and employability. The leading reason for limited ability to work and employability was restricted mobility or function of the affected limb along with time-consuming therapeutic modalities, pain and psychological stress. The most suitable job named was teacher and the most unsuitable job named was cook. As easements for return to work, early rehabilitation, self-management, coping strategies, patient education, employer’s goodwill and employer’s cooperation were reported. Furthermore, experts stressed the need for an adjustment of the legal framework as well as low-barrier and more therapy offers.

**Conclusions:**

Adjusted work demands seem to be of greater importance to support the ability to work and employability than recommendations for specific job profiles alone. Experts suggest an adjustment of the legal framework for affected patients, claiming a right for early rehabilitation as well as for life-long therapy. Even though some clinically useful conclusions may be drawn from this article, further research in the field is warranted.

## Introduction

Lymphedema is a chronic disease characterized by regional edematous swelling primarily affecting one or more limbs. In some cases, other body parts such as the trunk, the head or the genitals are affected as well. Swelling results from insufficient lymph transportation when tissue homeostasis is no longer sustainable. Lymphedema is an independent disease resulting from (i) a hereditary dysfunction or malformation of the lymphatic system (primary lymphedema) or (ii) from an acquired disease or disease-related therapeutic measure (secondary lymphedema). Primary lymphedema is for example related to fetal hygroma, Turner’s syndrome or similar inborn conditions [1]. On the other hand, secondary lymphedema is related to cancer (different entities), infections (e.g. filariasis), trauma and iatrogenic causes [1]. Lymphedema is a morbidity factor that lowers the function and mobility of the affected limb and causes paraesthesia. Even though prevalence rates in the general population vary over a wide range [2, 3] the problem is likely to be underestimated [4]. Recent prevalence estimates range from 1.33 per 1000 to 1.44 per 1000 [5, 6]; however, due to many different clinical detection methods for lymphedema that may not clearly be repeatable or valid, any reported epidemiological figure remains vague [7]. Due to the increasing number of cancer survivors, the sub-population of patients with cancer-related lymphedema is also increasing and requires special attention. Patients suffering from cancer-related lymphedema tend to have a reduced health-related quality of life (QoL) [8, 9]. Consequently, cancer patients often do not use their swollen arm (for example properly in the activities of daily living, as the function of the affected limb is limited) [10]. Additionally, the symptom lymphedema causes a significant increase in healthcare costs [11]. This challenging problem of lymphedema is mainly addressed by interventions such as complex decongestive therapy (CDT) as well as exercise and skin care [12]. As lymphedema is a chronic disease life-long therapy is required. A lack of information for patients as well as some health care professionals seems to worsen the problem of delayed or insufficient therapy [13–15]. The aim of the present study was to investigate the opinion of Austrian experts on factors influencing ability to work and employability in patients suffering from lymphedema.

## Method

Due to limited scientific evidence in the literature, the ideation process for this study was begun in May 2016. After conducting a literature search looking for articles investigating the influence of lymphedema on the ability to work and employability, 12 Austrian experts with longstanding expertise and efforts in lymphology were interviewed by using a self-administered questionnaire. All interviewees were active Austrian clinicians coming from 6 different specializations: physical medicine and rehabilitation (5), internal medicine/angiology (2), dermatology (1), internal medicine/hemato-oncology (1), surgery (1) and general medicine/family medicine (2). These experts were contacted via mail and asked to answer the 6 questions. Due to a broadly open question format, experts had the possibility to not just give short and straight answers but also to share some insights from their longstanding practice that might help to even find and define underrepresented fields of interest better.

The following questions were used:

1. Please name up to 5 professions, which are not suitable for patients suffering from lymphedema or for patients during lymphedema treatment.
2. Please name up to 5 professions, which are suitable for patients suffering from lymphedema or for patients during lymphedema treatment.
3. From your expertise do you think a lymphedema can restrict the ability to work/employability?
4. If yes, how can a lymphedema or lymphedema treatment restrict the ability to work/employability? Please name up to 5 reasons.
5. What makes it easier to stay at a workplace or what makes the return to work easier for patients already suffering from lymphedema?
6. What additional measures could support the stay at the workplace or the return to work for patients suffering from lymphedema?

**Fig. 1:**
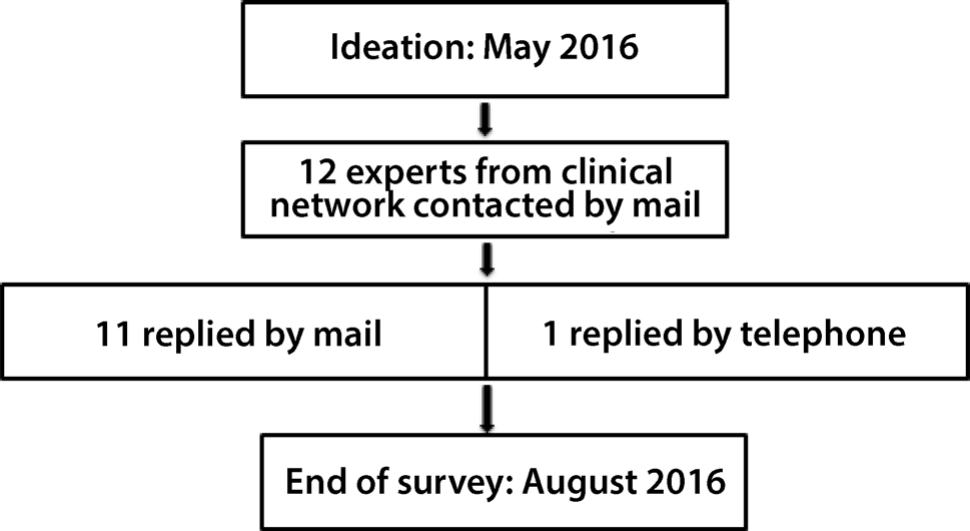
Flow chart: from ideation to the end of the survey

Fig. 1 shows the work-process from ideation until the end of the survey.

## Results

Out of 12 experts 11 replied via mail and 1 replied via telephone, resulting in a 100% reply rate. The last answers were received by 31 August 2016; however, questions 4, 5 and 6 were only sufficiently answered by 11 experts as in each case 1 expert left the answer box blank. An overview of the three most common answers is presented for each question in Table 1. The results for each question are presented in detail in Tables 2, 3, 4, 5 and 6.

**Table 1:** Overview of the three most common answers per question

Question		Three most common answers	
1	Unsuitable professions	(i)	Cook
		(ii)	Construction worker
		(iii)	Baker
2	Suitable professions	(i)	Teacher
		(ii)	Physicians, some specialties
		(iii)	Secretary
3	Restricted employability	100% consensus for “yes” (all experts agreed: that lymphedema can restrict workability/employability)	
4	Causes for restricted employability	(i)	Restriction of mobility or function
		(ii)	Time-consuming therapeutic modalities
		(iii)	Pain and psychological stress
5	Easements for return to work	(i)	Early rehabilitation
		(ii)	Self-management/coping strategies/patient education
		(iii)	Goodwill/employer’s cooperation
6	Additional measures	(i)	Legal framework/willingness of insurance providers (paid rehabilitation, right to have regular therapies)
		(ii)	Low-barrier and more therapy offers
		(iii)	Ergonomics and harmonious work intervals

Due to the open nature of questions, categories for questions 1 and 2 and for question 4–6 were defined (Table 1).

### Question 1.

Please name up to 5 professions that are not suitable for patients suffering from lymphedema or for patients during lymphedema related treatment.

Table 2 shows unsuitable professions sorted by frequency according to the experts’ answers. Due to the open questions, some experts commented on the question in general as well. Following these general answers, three categories were defined, focusing on specific activity-related conditions. The three categories were: physical strain (ergonomics, posture), jobs with increased risk of injury and climatic stress (e.g. heat, humidity). Some answers may be listed twice as they were not precisely assignable to one category.

The three most common answers for unsuitable professions were:

- (i) cook (42%),
- (ii) construction worker (33%),
- (iii) baker (33%).

**Table 2:** Unsuitable jobs sorted by frequency according to the expert opinions

Physical strain (ergonomics, posture) 25	Jobs with increased danger of injury 11	Climatic stress (heat, humidity) 6
Surgeon 2	Construction worker 4	Cook 5 Baker 4
Waiter 2	Lumberman 1	Cleaner (not specified) 3
Kindergarten teacher 2	Farmer 1	Pool attendant 2
Driver 2	Precision engineer 1	Furnace worker 1
Butcher 2	Athlete 1	Professions with exposure to dirt in general (not specified) 1
Roofer, +related jobs 2 Electrician 1	Health care professionals (not specified) 1	–
Shift worker 1	–	–
Road worker 1	–	–
Furniture remover 1	–	–
Postman 1	–	–
Retailer 2	–	–
Secretary 1	–	–
Lumberjack 1	–	–
Pilot 1	–	–
Steel worker 1	–	–
Typist 1	–	–
Physician1	–	–
Hairdresser 1	–	–
Theater nurse 1	–	–
Miner 1	–	–
Tennis teacher 1	–	–
Canoe driver (professional athletes in this sport) 1	–	–
Sales staff 1	–	–
Homemaker 1	–	–
Shop assistant 1	–	–

Attached numbers indicate how many out of 12 experts mentioned this job profile

### Question 2.

Please name up to 5 professions that are suitable for patients suffering from lymphedema or for patients during lymphedema related treatment.

Table 3 shows suitable professions sorted by frequency according to the experts’ answers. Due to the open questions, some experts commented on the question in general as well. As in answer 1 following these general answers, three categories were defined focusing on specific activity-related conditions. The three categories were: low physical strain, other and general unspecified answers. In the last category, some open answers are presented as well.

**Table 3:** Suitable jobs sorted by frequency according to experts’ opinion

Low physical strain 14	Others 4	General unspecified answers 4
Teacher 4	Physician (some specialties) 3	Executive position (change of body position possible)/management 2
Secretary 3	Psychotherapist/psychologist 2	Office work, if ergonomic 1
Accountant 2	Homemaker + work breaks (not specified) 2	Jobs with the possibility to raise the limbs/arms in general 1
Student 1	Light physical work (not specified) 1	“Everything that gives the freedom to move during work … and with low humidity and heat …” 1
Desk work 1	Service sector, not specified (traffic, art, media) 1	–
Part-time worker, limited 1	–	–
Retailer 1	–	–
Administrator 1	–	–
IT technician 1	–	–
Administrator 1	–	–
Call center 1	–	–
Government employee 1	–	–
Lawyer/notary 1	–	–
Architect 1	–	–

Attached numbers indicate how many out of 12 experts mentioned this job profile

The three most common answers for suitable professions were:

- (i) teacher (33%),
- (ii) physician, some specialties (25%),
- (iii) secretary (25%).

### Question 3.

From your expertise: Do you think a lymphedema can restrict the ability to work/employability?

This question revealed a 100% consensus for the answer “yes”. All experts agreed, that lymphedema can restrict the ability to work/employability.

### Question 4.

If yes: how can a lymphedema or lymphedema related treatment restrict the ability to work/employability? Please name up to 5 reasons!

Due to a broad variety of open answers, 6 categories were defined. Question 4 was only answered by 11 experts as 1 expert left the box blank; however, the majority of experts (65%) agreed that a restriction of mobility or function is the most common cause for restricted ability to work/employability in patients suffering from lymphedema or in patients receiving lymphedema-related treatment. Category 6 provides all other answers that did not fit into one of the previously defined categories. Table 4 shows causes for restricted ability to work/employability by experts’ opinion.

**Table 4:** The 5 categories of causes for restricted ability to work/employability in lymphedema patients and a summary of additional answers in the category “others”

Restriction of mobility or function	65%
Time-consuming therapeutic modalities	36%
Pain	36%
Psychological stress (stigmatization, self-image, existential fear etc.)	27%
Recurrent infections	18%
Others	Lymphedema-related complications Increased swelling during day 2 Requirement to be able to raise the limb/arm 1 Intolerance of heat 1 Restricted travel suitability (tropics e. g.) 1 Job-related stress 1 Decreased lymphedema stadium 1 Sedentary work, permanent 1 Increased limb volume + skin fibrosis 1 Restricted strength and endurance 1 Handicap for manual labour 1 Increased danger of injury 1 Sexual problems (genital lymphedema) 1 Partnership problems 1

The three most common causes for restricted ability to work/employability by experts’ opinion were:

- (i) restriction of mobility or function (65%),
- (ii) time-consuming therapeutic modalities (36%),
- (iii) pain and psychological stress (36%).

### Question 5.

What makes it easier to stay at a workplace or what makes the return to work easier for patients suffering from lymphedema?

Question 5 was only answered by 11 experts as 1 expert left the answer box blank; however, the answer most stressed by experts was early rehabilitation as the leading cause for improved return to work. The 4 other main answers are presented in Table 5 along with others – each of these answers were given only by 1 expert.

**Table 5:** Main causes for improved return to work due to the experts’ opinion

Early rehabilitation	27%
Self-management/coping strategies/patient education	27%
Goodwill/employer’s cooperation	27%
Improved patient information (about rehabilitation possibilities for outpatients and in-house patients, the effect of compliance on the outcome etc.)	18%
Restructuring the work field (no external service, retraining etc.)	18%
Others	State of the art therapies Legal right to receive rehabilitation and therapies Legal framework Reduced work time Work intervals Ergonomics Medical aids Easy accessibility Enjoyment of work

A summary of additional answers is presented in the category “others”

The three most common causes for improved return to work by experts’ opinion were:

- (i) early rehabilitation (27%),
- (ii) Self-management/coping strategies/patient education (27%),
- (iii) Goodwill/employer’s cooperation (27%).

### Question 6.

What additional measures could support the stay at the workplace or the return to work for patients suffering from lymphedema?

Question 6 was only answered by 11 experts as 1 expert left the answer box blank and 15 categories were defined for question 6. A summary is presented in Table 6. In the open answer format the legal framework was the most emphasized measure in order to provide a better support for return to work.

**Table 6:** Additional measures that could support the stay at the workplace for lymphedema patients by experts’ opinion

Legal framework/willingness of insurance providers (paid rehabilitation, right to have regular therapies)	36%
Low-barrier and more therapy offers	36%
Ergonomics and harmonious work intervals	36%
Awareness/information, better information for colleagues and superiors	27%
Possibility of climatic adaption	27%
Reduced and flexible work time	27%
Early rehabilitation/adequate rehabilitation	18%
Workshops/information events	18%
Changed laws/willingness of insurance providers	18%
Job-related measurements (sanitary adjustments of work place e. g.)	18%
Professional support for job reintegration	18%
Retraining	9%
Adequate exploitation of financial, temporal and psychological resources	9%
Self-management	9%
Sheltered employment due to partial handicap (more vacation days etc.) and support for employers to reintegrate lymphedema patients	9%

The three most important additional measures by experts’ opinion were:

- (i) legal framework/willingness of insurance providers (paid rehabilitation, right to have regular therapies),
- (ii) low-barrier and more therapy offers,
- (iii) ergonomics and harmonious work intervals.

## Discussion

The aim of this study was to provide a better insight in existing knowledge and clinical expertise by conducting an expert survey and a literature review. Furthermore, this survey was undertaken in order to help generate future research questions in the field. Recent scientific literature dealing with the influence of lymphedema on employability and the ability to work is scarce; however, some significant parallels of existing literature with the presented experts’ opinion can clearly be drawn. Indications in the current literature show that jobs requiring heavy physical work, heavy lifting or similar physical strain are unsuitable for patients suffering from lymphedema [14–16]. Likewise, interviewed experts as well described jobs related with physical strain, increased danger of injury or climatic stress as not suitable. Nevertheless, in some cases specific job profiles may differ in the scientific literature as well as among experts’ answers. For example, the job profile secretary was explicitly mentioned by 1 expert as not suitable, by 3 experts as suitable. In comparison, the profession secretary was described in a study by Fu as partially suitable [14]. A possible explanation for these seemingly contradictory statements may be the imprecisely defined work demands that a secretary may need to fulfil in different work environments; therefore, it seems to be important not only to name specific job profiles as suitable or not suitable but to generally define suitable and unsuitable work demands. This approach may also help better customization of individually tailored return to work reintegration processes both for affected employees as well as for employers. Furthermore, professions requiring heavy physical work and heavy lifting are not recommended for patients suffering from lymphedema [14–16]. Likewise, interviewed experts stressed low physical strain to be the primarily important condition for suitable job profiles. Overlapping examples were teacher and manager/executive.

Boyages et al. showed a significant negative influence of lymphedema on work and career [13] supporting experts’ opinion that lymphedema can restrict the ability to work/employability. Considering reasons for reduced ability to work/employability resulting from lymphedema, restricted mobility or function of the affected limb is named as one of the leading reasons both in the literature as well as a result of the expert survey [14, 15]. Additionally, psychosocial reasons came into focus. Johansson et al. mentioned an altered self-image as ugly and unfeminine in relation to the compression sleeve as an important limiting factor [15]. Likewise, the experts mentioned stigmatization and altered self-image. Additionally, Fu mentioned emotional stress due to constant job worries and the visible sign of limitation or disability as relevant factors [14]. Greenslade and House described in a qualitative study the patient’s perspective regarding lymphedema and stated fear of loss, anxiety and sadness – also due to limited information about the disease and treatment options – as relevant factors for restricted ability to work/employability [17]. Psychological stress was the fourth most common answer from interviewed experts in a similar manner.

Considering suggestions for improvement of the existing healthcare environment, both the interviewed experts as well as Boyages et al. stressed the need for a change of the legal framework [13]. In particular attention should be drawn to lymphedema as a chronic versus an acute disease in repect to the labour law. Furthermore, the alteration of the legal framework and the willingness of insurance providers were emphasized by experts. They also claimed that patient should have the right to receive correct lymphedema treatment. Another definite parallel of suggestions for additional measures was the reduction of work time and the flexibility of work intervals [15]. Finally, improved information for patients as well as for medical professionals about lymphedema as a chronic condition and therefore a need for lifelong therapy was repeatedly stressed both in the literature as well as from the interviewed experts [14].

## Conclusion

Adjusted work demands seem to be of greater importance to support the ability to work/employability than recommendations for specific job profiles only. Experts in accordance with the literature, suggest an adjustment of the legal framework for affected patients, claiming a right for early rehabilitation as well as for lifelong therapy. Even though some clinically useful conclusions may be drawn from this article, literature about this highly relevant, clinical issue remains scarce, warranting further research in the field.
